# Development of Pretreatment Approaches for Authentic Representation of Tea Infusion Aroma

**DOI:** 10.3390/foods14162759

**Published:** 2025-08-08

**Authors:** Mingming Zhang, Zhihui Feng, Fang Wang, Jianxin Chen, Yifan Li, Yuqiong Chen, Junfeng Yin

**Affiliations:** 1 Key Laboratory of Tea Biology and Resource Untilization, Ministry of Agriculture, Tea Research Institute, Chinese Academy of Agricultural Sciences, Hangzhou 310008, China; zhangmingming@tricaas.com (M.Z.);; 2 National Key Laboratory for Germplasm Innovation and Utilization for Horticultural Crops, College of Horticulture & Forestry Sciences, Huazhong Agricultural University, Wuhan 430070, China; 3 Graduate School of Chinese Academy of Agricultural Sciences, Beijing 100081, China

**Keywords:** headspace solid phase microextraction, ions in water, brewing aroma, sensory fidelity

## Abstract

Appropriate aroma extraction methods are crucial prerequisites for accurately and objectively characterizing the authentic aroma profile of samples. Purified water and ionized water were used as brewing water, and the effects of different tea-to-water ratios, extraction temperatures, and extraction times on the aroma authenticity and component enrichment of tea infusions were compared. The conditions of a tea-to-water ratio of 1 g:10 mL, extraction at 30 °C for 30 or 45 min were identified as the optimal parameter range, which could maximize the enrichment of aroma while maintaining fidelity. The cosine value of the aroma attribute scores between the optimal parameter set and the control group (tea brewed at 1 g:10 mL ratio for 4 min) exceeded 0.979, and the correlation coefficient surpassed 0.828. Test evaluation results indicate the method had good reproducibility and effectively highlighted the differential impacts of ionic content in brewing water on tea aroma constituents. This approach effectively solved the problem of sensory distortion caused by conventional high-temperature and long-duration extraction, enabling precise analysis of how water quality authentically influences tea infusion aroma characteristics.

## 1. Introduction

Aroma has long been a focal and trending area in flavor chemistry research, with extraction and detection techniques now relatively well-established. Headspace solid-phase microextraction (HS-SPME) is widely employed for aroma extraction due to its simplicity, solvent-free nature, and excellent thermal sensitivity [1,2,3]. Gas chromatography–mass spectrometry (GC-MS), as a mature analytical technique, is frequently combined with HS-SPME for aroma studies owing to its minimal sample requirement, high sensitivity, rapid analysis, and strong identification capabilities [4,5,6]. To achieve efficient and comprehensive aroma enrichment, previous studies have optimized HS-SPME conditions, identifying key factors such as fiber type, extraction temperature, extraction time, and salt ion concentration. However, optimal parameters vary depending on the analytes or experimental objectives. For instance, Yin et al. [7] found that for a chestnut-like aroma of green tea, the best adsorption was achieved using a DVB/CAR/PDMS fiber at 50 °C for 50 min, with a fixed tea-to-water ratio of 1 g: 10 mL and an electrolyte concentration of 1–3 mg·mL^−1^. In contrast, Lv et al. [8] reported that for West Lake Longjing tea, optimal adsorption occurred under electrolyte-free conditions, using a DVB/CAR/PDMS fiber at 60 °C for 30 min with a tea-to-water ratio of 1 g: 50 mL. However, Wang et al. [9] demonstrated that for Dianhong tea, the ideal conditions involved a DVB/PDMS fiber at 70 °C for 60 min, an electrolyte concentration of 0.33 g·mL^−1^, and a tea-to-water ratio of 1 g: 3 mL. Most existing studies prioritize maximizing total peak area or the peak area of key active compounds, often favoring high-temperature, long-duration extraction (e.g., 60 °C for 60 min [10], 70 °C for 55 min [11] or 60 min [12], 80 °C for 60 min [13]) Nevertheless, these processing conditions risk altering sensory profiles, especially in green tea—an aspect that has not been systematically evaluated. Longjing tea, renowned for its emerald color, elegant appearance, mellow aroma, and refreshing taste, is universally acknowledged as the typical representative of green tea. Its distinctive fragrance becomes particularly intense and persistent through high-temperature roasting processes, making it highly prized by consumers and extensively studied by researchers. Wang et al. [14] detected 151 volatile compounds across 29 Longjing tea samples, among which 14 key aroma-active compounds were identified through combined gas chromatography–olfactometry (GC-O) analysis, odor activity value (OAV) calculation, and aroma recombination experiments. The identified compounds include 2-methyl butyraldehyde, dimethyl sulfoxide, heptanal, benzaldehyde, 1-octen-3-ol, (E,E)-2,4-heptadienal, phenylacetaldehyde, linalool oxide I, (E,E)-3,5-octadien-2-one, linalool, nonanal, methyl salicylate, geraniol, and β-ionone. Notably, β-Ionone, 1-methylnaphthalene, naphthalene, 2,4-nonadienal, (E)-2-nonenal, and decanal exhibited exceptionally high OAVs (>1000) and were demonstrated to be primary contributors to the characteristic aroma profile of Longjing tea. Zhu et al. [15] employed GC-O and OAV analysis to characterize the aroma profiles of Longjing teas across different grades and geographical origins. (E,E)-2,4-heptadienal, 3-methylbutanal, dimethyl sulfide, linalool, 2-methylbutanal,α-terpineol, geraniol, dimethyl sulfoxide, furaneol, and cis-jasmone were identified as the primary aroma compounds and (E,E)-2,4-heptadienal, 3-methylbutanal, geraniol, dimethyl sulfide, 2-methylbutanal were considered as the critical aroma-active compounds defining Longjing tea. Lin et al. [16] identified these 10 compounds (linalool, nonanal, safranal, β-farnesene, myrcene, (Z)-3-hexenyl hexanoate, hexyl hexanoate, geranyl acetone, β-ionone, and an unknown compound) as making significant contributions to Longjing tea’s aroma profile, particularly linalool, nonanal, (Z)-3-hexenyl hexanoate, and β-ionone. In contrast, Li et al. [17] reported a different top-10 set of aroma contributors for Longjing tea infusion, comprising dimethyl sulfide, 3-methylbutanal, 2-methylbutanal, hexanal, octanal, linalool, nonanal, decanal, 3-methylnonane-2,4-dione, and β-ionone. It is evident that the aroma compounds 3-methylbutanal, 2-methylbutanal, linalool, nonanal, decanal, and β-ionone represent particularly crucial components of Longjing tea’s characteristic flavor profile.

Water is a critical medium for tea quality formation and transformation, with different water qualities yielding distinct tea liquor characteristics and nutritional profile [18,19,20,21]. While numerous studies highlight the impact of water quality on aroma based on sensory evaluations, few have investigated its influence on aroma composition [17], and optimized extraction conditions for aroma analysis remain scarce. Notably, no study has explored whether high-temperature, prolonged extraction affects the true aroma profile of tea infusion brewed with different water quality. However, establishing an appropriate aroma extraction method is essential for efficient and accurate preparation of aroma compounds, enabling precise analysis of water quality’s impact on tea aroma. Research indicates that different tea categories exhibit varying sensitivities to water quality [22]. Green tea demonstrates particularly high sensitivity to ionized water [23], and premium-grade green teas generally exhibit higher sensitivity to water quality variations compared to conventional-grade green teas [24]. Green tea tends to develop a cooked aroma character when brewed with high-ion-content water, and this effect is markedly exacerbated under prolonged high-temperature conditions [23]. In other words, the aromatic profile of green tea may undergo transformations during extraction when brewed with different water quality. Consequently, investigating optimal aroma extraction conditions for green tea infusions prepared with varying water quality becomes particularly imperative.

This study employs HS-SPME/GC-MS to compare extraction conditions for aroma analysis of Longjing tea brewed with different water qualities, aiming to identify parameters that authentically represent green tea soup aroma and maximize the enrichment of aroma components. The findings are expected to provide theoretical and technical guidance for future research on water quality’s influence on tea aroma substances.

## 2. Materials and Methods

### 2.1. Materials and Chemicals

Based on extensive preliminary tasting results, premium Longjing tea (Hangzhou Longguan Industrial Co., Ltd., Hangzhou, China), which is highly sensitive to water quality, was selected as the experimental tea. Commercially available water with high ion content (total dissolved solids-TDS = 500 mg/L) was used as the typical water sample (purchased from an online platform), while purified water (TDS = 3.92 mg/L) (Hangzhou Wahaha Group Co., Ltd., Hangzhou, China) served as the control for experiments. The main reagents included 34 n-alkanes (C7–C40, ANPEL Laboratory Technologies Inc., Shanghai, China) and an internal standard substance (p-Xylene-d10, Shanghai Aladdin Biochemical Technology Co., Ltd., Shanghai, China).

### 2.2. Equipment and Apparatus

Equipment included an SQP electronic balance (Sartorius Scientific Instruments Co., Ltd., Beijing, China); DF-101S thermostatic heating magnetic stirrer (Yuhua Instrument Co., Ltd., Gongyi, China); 7890B-7000C gas chromatography-mass spectrometer (Agilent Technologies, Santa Clara, CA, USA); SPME handle with a DVB/CAR/PDMS fiber (50/30 μm, 2 cm, Supelco, Bellefonte, PA, USA); and 20 mL and 100 mL headspace vials with caps (ANPEL Laboratory Technologies Inc., Shanghai, China).

### 2.3. HS-SPME Optimization Design and Procedures

#### 2.3.1. HS-SPME Optimization Design

The DVB/CAR/PDMS (divinylbenzene/carboxen/polydimethylsiloxane) coating, which combines polar and non-polar properties and can adsorb a broader range of aromatic compounds [7,25,26], was employed for optimization experiments. No electrolyte was added in this experiment to prevent interference from water ions on aroma perception. A single-factor method was employed to optimize three key parameters affecting aroma extraction: tea-to-water ratio, extraction temperature, and extraction time (Table 1). The factor levels were set based on suitable conditions reported in the literature, with a fixed combination of 1 g:50 mL tea-to-water ratio, 30 °C extraction temperature, and 30 min extraction time [17]. The variable control method was used to incrementally adjust single-factor levels to identify a method that efficiently and accurately extracts the complete aroma profile. When investigating the tea-to-water ratio, the extraction temperature (30 °C) and time (30 min) were held constant, while the ratio was varied at 1 g:50 mL, 1 g:20 mL, and 1 g:10 mL. When examining extraction temperature, the tea-to-water ratio (1 g:50 mL) and extraction time (30 min) remained unchanged, with temperatures tested at 30 °C, 45 °C, and 60 °C. When assessing extraction time, the tea-to-water ratio (1 g:50 mL) and temperature (30 °C) were fixed, and durations were adjusted to 30 min, 45 min, and 60 min.

#### 2.3.2. HS-SPME Procedures

Longjing tea was brewed for 4 min at the designated tea-to-water ratio, rapidly filtered, and cooled to room temperature (25 °C). Then, 50 mL of the tea infusion was transferred to a 100 mL headspace vial containing a stir bar, spiked with an internal standard (2.7 mg/L, 10 μL p-xylene-d_10_), and sealed. The headspace vial was equilibrated for 5 min in a magnetic stirrer at the target temperature. The SPME fiber was inserted into the headspace 2 cm above the tea infusion and exposed for the specified time. After extraction, the fiber was immediately inserted into the GC-MS inlet for thermal desorption (5 min). Each sample was performed in triplicate.

### 2.4. Aroma Detection and Analysis

#### 2.4.1. Aroma Detection

**Chromatographic conditions:** Separation was performed using a DB-5MS column (30 m × 250 μm × 0.25 μm) with high-purity helium (purity > 99.99%) as the carrier gas. A straight ultra-inert liner (GHAJ-5190-4048, 78.5 mm × 0.75 mm ID, 35 μL) was used for SPME injection. The inlet temperature was set at 250 °C, and the column flow rate was 1.0 mL/min in splitless mode. For extraction optimization experiments, the temperature program was an initial temperature of 40 °C for 2 min, ramped at 4 °C/min to 120 °C, then rising to 260 °C at a rate of 30 °C/min, and held for 5 min. For repeatability validation experiments, the program was adjusted to 40 °C, held for 5 min, ramped at 4 °C/min to 120 °C, then at 10 °C/min to 260 °C, and held for 6 min. To prevent carryover, blank runs were performed after each sample analysis to clean the fiber and column.

**Mass spectrometric conditions:** Electron ionization (EI) was used with an ion source temperature of 230 °C and electron energy of 70 eV. The quadrupole temperature was set at 150 °C, and the transfer line temperature was 280 °C. The mass scan range was 35–350 amu in full-scan mode.

#### 2.4.2. Aroma Analysis

The qualitative analysis of the volatile components was processed by Agilent MassHunter Workstation Software Unknowns Analysis. The compounds were tentatively identified by comparing the obtained spectra with those from National Institute of Standards and Technology (NIST14) when the similarity was above 80%. Retention indices (RIs) were calculated based on the linear equation of n-alkanes (C7–C40) and compared to the matched compounds in the NIST library or literature (RI deviation ≤ 30). We utilized laboratory-identified dimethyl ether as a reference standard, incorporating its experimentally determined retention time (RT) and retention index (RI) to supplement the n-alkane series data for extending the linear calibration range prior to C7.

For optimization experiments, relative peak area was used to calculate compound content [27]:

(1) $$C_{i}(\%)=\frac{P_{t}}{P_{i}}\times 100$$

where *C_i_* is the concentration of the target compound (%); *P_t_* is the target peak areas; *P_i_* is the internal standard peak area.

For validation experiments, compound content was calculated according to the internal standard method:

(2) $$C_{i}(\mu g/kg)=\frac{C_{is}\times A_{i}}{A_{is}}$$

where *C_i_* is the concentration of the target compound (μg/kg); *C_is_* is the internal standard concentration (μg/kg); *A_i_* and *A_is_* are the peak areas of the target and internal standard, respectively.

### 2.5. Sensory Fidelity Comparison and Verification

#### 2.5.1. Sensory Fidelity Comparison

The sensory evaluation of tea infusion was performed according to the National Standard of China (GB/T23776-2018) [28]. The sensory attributes were independently scored by 7 professional tea tasters on a 10-point scale (0: imperceptible; 1–2: faint; 3–4: detectable; 5–6: moderate; 7–8: strong; 9–10: intense), with the attributes such as tender aroma, chestnut aroma, sweet aroma, stuffy and ripeness, and metallic off-flavor. The score results were calculated as the mean value after removing the highest and lowest scores. A radar chart was generated to compare aroma profiles, and conditions yielding the closest match to the control (CK, tea brewed at 1:50 ratio for 4 min) were selected for further validation.

#### 2.5.2. Sensory Fidelity Verification

Panelists rated the similarity between selected samples and CK on a 5-point scale: 5 (81–100% match), 4 (61–80% match), 3 (41–60% match), 2 (21–40% match), 1 (1–20% match), or 0 (0%). Parallel experiments were conducted for purified water and ion-rich water groups. For the purified water group tea-to-water ratio, Longjing tea was brewed with 100 °C purified water at ratios of 1 g:50 mL, 1 g:20 mL, and 1 g:10 mL for 4 min, filtered, cooled, and extracted at 30 °C for 30 min. For the extraction temperature, tea (1 g:50 mL ratio) was extracted at 30 °C, 45 °C, and 60 °C for 30 min. For the extraction time, tea (1 g:50 mL ratio) was extracted at 30 °C for 30, 45, and 60 min. We placed the samples that underwent longer extraction times in the water bath first, to ensure the processing of the three different times ended simultaneously for synchronous evaluation. All samples were equilibrated at 25 °C for 10 min before evaluation.

During sensory validation experiments under both maximum aroma enrichment conditions and optimal fidelity conditions, a control group was established using a tea-to-water ratio of 1 g:10 mL with 4 min of brewing time. The evaluation principles for sensory attribute scoring and similarity assessment remained consistent with the aforementioned criteria.

### 2.6. Feasibility and Application Effectiveness Analysis of Pretreatment Methods

Cosine similarity and correlation coefficients are used as quantitative indicators of aroma similarity, with values closer to 1 indicating higher similarity [7]. Internal standards were used to monitor extraction and detection consistency. Retention time and peak area variations were required to be ≤1.5% [7] and ≤25% [27], respectively. Optimal conditions were selected based on good fidelity, high aroma content, and well reproducibility.

To evaluate the practical application effect of high-fidelity extraction conditions, we analyzed the aroma components extracted under both maximum extraction yield conditions and high-fidelity conditions. This comparison aimed to determine whether they exhibit distinguishable differences in aroma composition profiles, thereby validating the performance of the sensory evaluation results. Additionally, ionized water diluted to half its original TDS concentration (from 500 mg/L to 250 mg/L) was tested under the optimal extraction conditions to evaluate the method’s discriminative power for different ion water.

### 2.7. Statistical Analysis

Each independent experiment was performed in triplicate. The mean, deviation, cosine similarity was calculated by Microsoft Excel 2019 software. The statistical significance analysis (Duncan’s test, *p* < 0.05) and correlation analysis were used IBM SPSS Statistics 27. The radar charts were plotted with Origin 2021 software and the histograms were plotted with GraphPad Prism 9 software. Cluster analysis and partial least square discriminate analysis (PLS-DA) were conducted with SIMCA 13.0 software. Cluster analysis refers to a family of algorithms that partition a dataset into distinct subgroups (clusters), where objects within the same cluster exhibit higher similarity compared to those in different clusters. PLS-DA is a commonly used statistical method to determine how subjects are classified based on the observed or measured values of several variables. The closer the R^2^ and Q^2^ of PLS-DA are to 1, the stronger the explanatory and prediction ability of PLS-DA.

## 3. Results Analysis

### 3.1. Analysis of Aroma Extraction Efficiency

#### 3.1.1. Comparison of the Total Peak Area of Aroma Extraction

**Figure 1:**
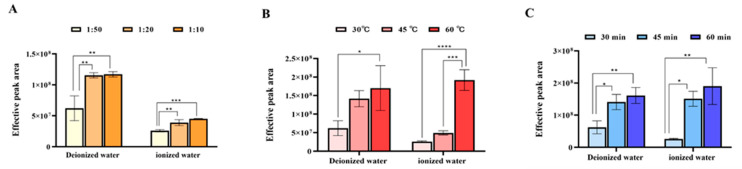
Comparison of total effective peak areas under different extraction conditions of tea aroma compounds (“*” indicates the level of significance. “*” means *p* < 0.05; “**” means *p* < 0.01; “***” means *p* < 0.001; “****” means *p* < 0.0001). (**A**) Tea-to-water ratio(“1:50” means 1 g:50 mL; “1:20” means 1 g:20 mL; “1:10” means 1 g:10 mL); (**B**) Extraction temperature; (**C**) Extraction time.

When extraction reaches equilibrium among solid, liquid, and gas phases, the adsorption capacity of the fiber for volatile compounds is directly proportional to their concentration in the liquid sample [29]. Generally, higher tea-to-water ratios yield greater aroma compound concentrations [12]. As shown in Figure 1A, the effective total peak area (after excluding silicone bleed and other interference peaks) increased significantly with a higher tea-to-water ratio, reaching its maximum at a 1 g:10 mL ratio, indicating optimal extraction efficiency. Extraction temperature determines the partition coefficient of aroma compounds between the fiber and the matrix. Higher temperatures generally increase compound volatility and reduce equilibrium time. However, excessively high temperatures may elevate water vapor content, impair fiber adsorption, or trigger undesirable chemical reactions. Figure 1B demonstrates that the tested temperature range was appropriate, with no inhibitory effects observed. The effective peak area rose significantly with temperature and peaked at 60 °C, indicating the optimal extraction temperature for the maximum chemical yield. Extraction time affects reproducibility and efficiency. Prolonged time initially increases enrichment but may plateau or decline due to competitive adsorption. Figure 1C shows that the effective peak area grew with time, rising rapidly from 30 to 45 min and more slowly thereafter. The maximum extraction efficiency was attained at 60 min, indicating the optimal extraction duration for the maximum chemical yield.

In summary, both water types exhibited consistent trends: higher concentration of tea infusion, higher temperatures, and longer extraction times enhanced aroma enrichment. The optimal conditions for maximum chemical yield were a 1 g:10 mL tea-to-water ratio, extracted for 60 min under 60 °C.

#### 3.1.2. Comparison of the Peak Area of Important Aroma Substances

**Figure 2:**
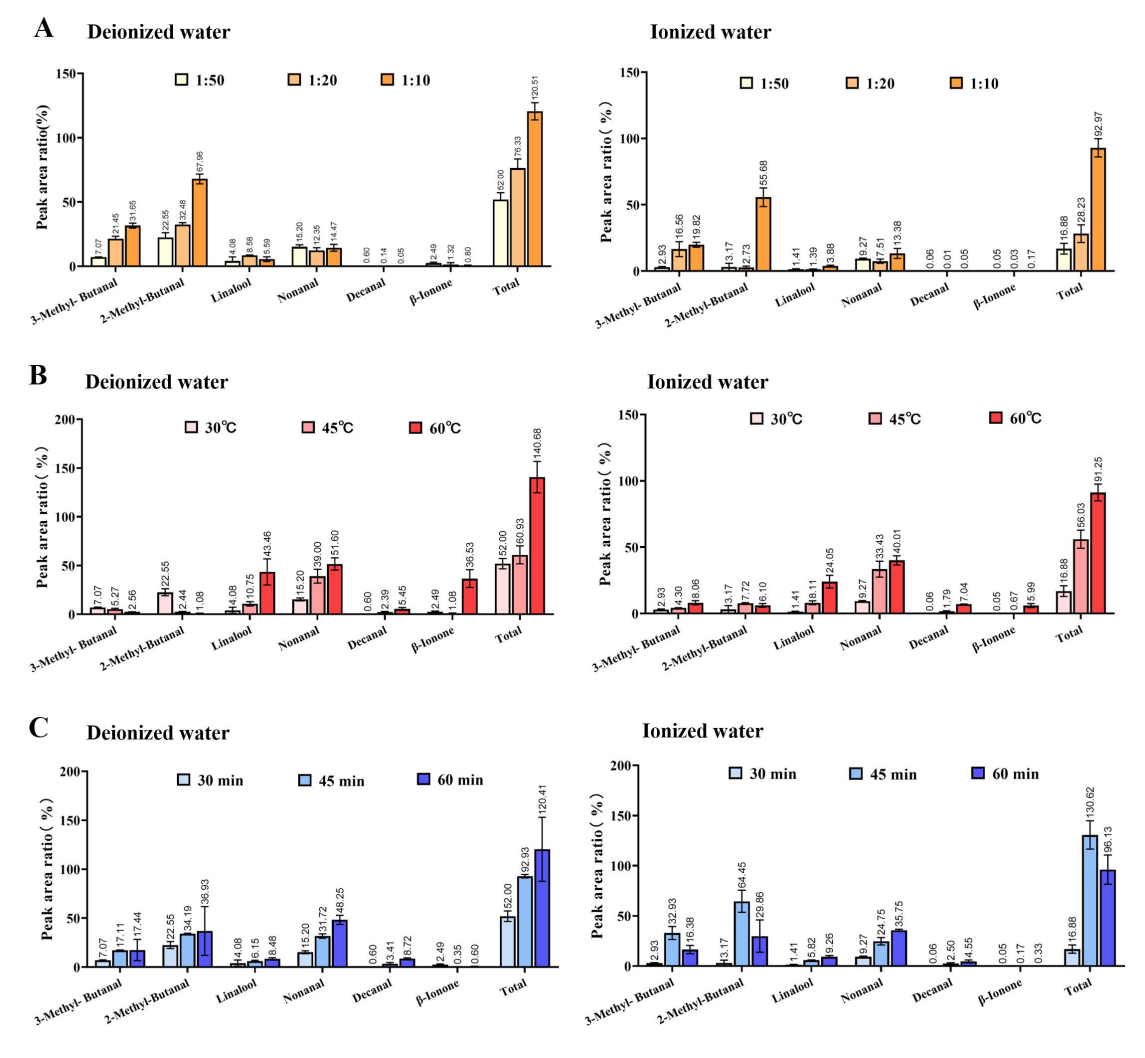
Comparison of relative contents of key aroma compounds under different extraction conditions of tea aroma compounds. (**A**) Tea-to-water ratio (“1:50” means 1 g:50 mL; “1:20” means 1 g:20 mL; “1:10” means 1 g:10 mL); (**B**) Extraction temperature; (**C**) Extraction time.

3-Methylbutanal, 2-methylbutanal, linalool, nonanal, decanal, and β-ionone have always been identified as critical aroma compounds in Longjing tea [14,15,16,17]. The effects of extraction conditions on these compounds were further analyzed. Figure 2A reveals that in purified water, 3-methylbutanal, 2-methylbutanal, and their total content increased with higher tea-to-water ratios, while linalool peaked at a 1 g:20 mL ratio before declining. Nonanal fluctuated slightly, whereas decanal and β-ionone decreased. In ion-rich water, 3-methylbutanal and total content rose steadily, while 2-methylbutanal and others remained stable until the 1 g:10 mL ratio, where all compounds surged. At tea-to-water ratios of 1g:10 mL, total aroma content peaked at 92.97% (ion-rich water) and 120.51% (purified water). Further comparative analysis revealed that the variation trends in 3-methylbutanal and 2-methylbutanal content with changing tea-to-water ratios were similar between the two systems, whereas other compounds exhibited divergent trends. This discrepancy may be attributed to ionic interference effects present in the aqueous ion system. As short-chain aliphatic aldehydes, 3-methylbutanal and 2-methylbutanal are derived from the Strecker degradation of leucine and isoleucine, respectively. The observed increase in their concentrations is likely due to the progressive accumulation of dissolved substrate precursors in the tea infusion. The absence of an increasing trend for other compounds in the pure water system may be attributed to either intrinsic concentration-dependent inhibition effects limiting their release or stability, or reduced selective adsorption capacity of SPME fibers at higher concentrations, ultimately resulting in measured content depletion.

Figure 2B shows that in purified water, 3-methylbutanal and 2-methylbutanal decreased with temperature, while other compounds and total content increased. High-temperature conditions generally promote the volatilization of aromatic compounds. However, the thermal instability of amino acids at relatively high temperatures may lead to degradation of their precursor substrates required for short-chain aliphatic aldehyde formation, consequently reducing the concentrations of 3-methylbutanal and 2-methylbutanal. In ion-rich water, all compounds and total content except 2-methylbutanal rose. The distinctive behavior of 2-methylbutanal content may be attributed to ionic interference effects, potentially leading to either its reduced concentration or diminished volatility, though the exact mechanism requires further investigation. When the extraction temperature was 60 °C, the total concentration of volatile compounds peaked in both systems, reaching 91.25% in ionized water and 140.68% in pure water. Figure 2C indicates that in purified water, all compounds except β-ionone increased over time. β-ionone is formed through oxidative degradation or enzymatic cleavage of carotenoids, or alternatively released via hydrolysis of β-ionone glucosides. While its concentration would theoretically increase with prolonged extraction time, the observed plateau after 30 min of extraction may be attributed to the declining adsorption efficiency of SPME fibers. In ion-rich water, 3-methylbutanal, 2-methylbutanal, and total content peaked at 45 min before declining, while others rose steadily, which likely results from the combined effects of ionic interactions and SPME fiber adsorption dynamics. In summary, purified water’s total content peaked at 60 min (reached 120.41%), whereas ion-rich water’s maximum (reached 130.62%) occurred at 45 min, dropping to 96.12% by 60 min.

Thus, key aroma compounds responded variably to extraction conditions, with divergent trends between water types. Overall, the optimal combination for maximum chemical yield was a 1g:10 mL tea-to-water ratio, with extraction at 60 °C for 60 min.

### 3.2. Fidelity Assessment of Extracted Aroma Profiles

#### 3.2.1. Fidelity Evaluation Under Maximum Aroma Enrichment Conditions

**Figure 3:**
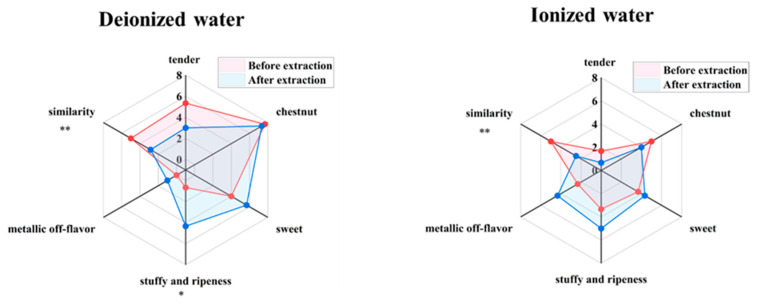
Aroma fidelity evaluation under maximum enrichment extraction conditions of tea aroma compounds (“*” indicates the level of significance. “*” means *p* < 0.05; “**” means *p* < 0.01).

The aforementioned results indicated that a tea-to-water ratio of 1 g:10 mL, an extraction temperature of 60 °C, and an extraction time of 60 min gathered the highest aroma content. To further investigate the fidelity of the aroma, tea infusions prepared under the condition using pure water and ionized water were compared with their respective counterpart CK (tea brewed at 1g:10 mL ratio for 4 min) in terms of aroma attributes and similarity. As shown in Figure 3, when evaluated against their respective control groups (CK, scored out of 5), the similarity scores for the pure water system and ionized water system were 2.84 and 2.50, respectively, both showing a significant decline (*p* < 0.01). In the pure water system, the scores for tender aroma and chestnut aroma decreased by 2.34 and 0.33 points, respectively, while sweet aroma, stuffy and ripeness flavor, and metallic off-flavor increased by 1.67, 3.67, and 1.00 points, respectively. In the ionized water system, tender aroma and chestnut aroma decreased by 1.00 and 1.00 points, respectively, whereas sweet aroma, stuffy and ripeness flavor, and metallic off-flavor increased by 0.67, 1.66, and 2.00 points, respectively. Under these extraction conditions, both systems exhibited noticeable changes in aroma profiles, characterized by a reduction in tender and chestnut aromas and an increase in sweet aroma, stuffy and ripeness flavor, and metallic off-flavor. Notably, the change in stuffy and ripeness flavor in the pure water system reached a significant level (*p* < 0.05).

#### 3.2.2. Exploration of High-Fidelity Aroma Extraction Conditions

**Figure 4:**
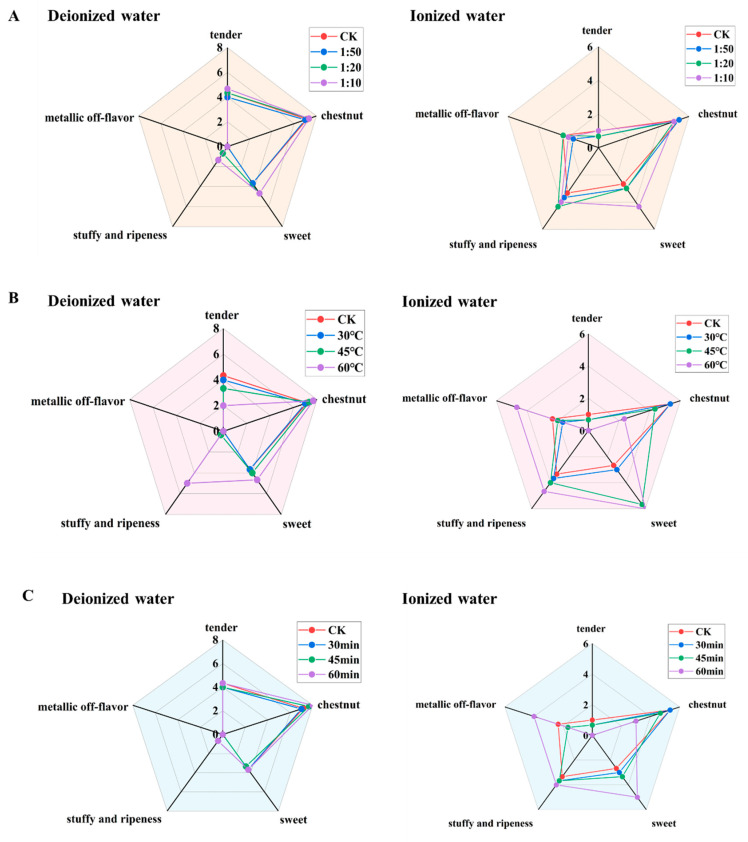
Comparison of aroma fidelity of tea infusion under different extraction conditions. (**A**) Tea-to-water ratio (“1:50” means 1 g:50 mL; “1:20” means 1 g:20 mL; “1:10” means 1 g:10 mL); (**B**) Extraction temperature; (**C**) Extraction time.

The above findings demonstrated that high-temperature, long-duration extraction conditions can easily distort tea aroma, necessitating the identification of more suitable extraction parameters to mitigate such adverse effects. Based on the established aroma extraction conditions, sensory fidelity was compared. The control groups for pure water and ionized water systems were brewed for 4 min at a tea-to-water ratio of 1 g:50 mL. The effects of varying tea-to-water ratios, extraction temperatures, and durations on sensory fidelity were investigated, as illustrated in Figure 4.

Figure 4A reveals that as the tea-to-water ratio increased, the aroma profiles of both systems remained relatively similar before and after extraction, with changes in each individual aroma attributes not exceeding 1.67 points. This suggested that a tea-to-water ratio within 1g:10 mL had minimal impact on the overall aroma characteristics. Figure 4B shows that at an extraction temperature of 30 °C, changes in aroma attributes were limited to 0.67 points, and the aroma profiles of both systems closely resembled their respective CKs. At 45 °C, the pure water system exhibited minor changes (all within 1.00 point), while the ionized water system began to deviate from its CK, particularly due to a sharp increase (3.00 point) in sweet aroma. At 60 °C, both systems showed significant divergence from their CKs, with pronounced changes in aroma attributes. For instance, in the pure water system, tender aroma decreased by 2.34 points, and stuffy and ripeness flavor increased by 5 points. In the ionized water system, chestnut aroma decreased by 3.00 points, while sweet aroma and metallic off-flavor increased by 3.33 and 2.33 points, respectively. Even with a short extraction time at 30 min, high temperatures markedly altered the aroma profiles, intensifying stuffy and ripeness flavor in the pure water system and metallic off-flavor and sweet aroma in the ionized water system. Thus, an extraction temperature of 30 °C was more conducive to preserving the authentic aroma of both systems. Figure 4C indicates that prolonged extraction time had little effect on the pure water system, whereas the ionized water system showed noticeable changes only after 60 min. For extraction times of 30 or 45 min, changes in each aroma attributes were within 0.67 points, indicating a negligible impact on fidelity. At 60 min, the pure water system exhibited a slight increase in chestnut aroma (0.83 points) and stuffy and ripeness flavor (0.67 points), while the ionized water system showed a significant reduction in chestnut aroma (2.34 points) and increases in sweet aroma (2.33 points) and metallic off-flavor (1.66 points). Therefore, an extraction time of 30–45 min minimized changes in overall aroma profiles.

In summary, an extraction temperature of 30 °C and a duration of 30–45 min enhanced the fidelity of pure/ionized water-infused aroma, while the tea-to-water ratio has a less significant impact.

#### 3.2.3. Validation of Olfactory Authenticity

**Figure 5:**
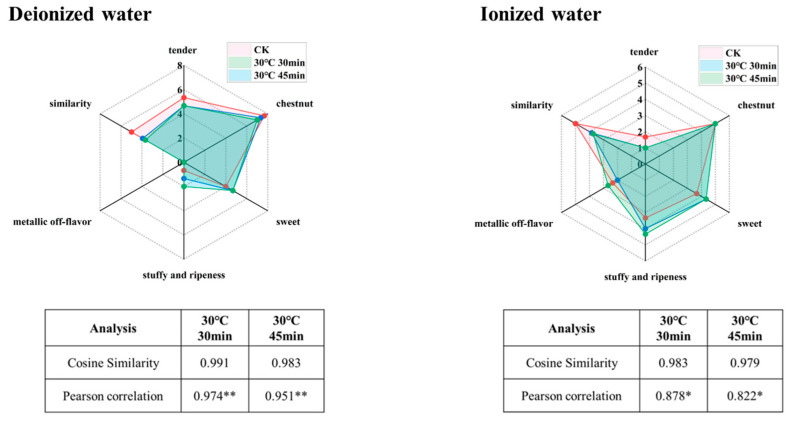
Sensory validation of extraction conditions with optimal fidelity of tea infusion (“*” indicates the level of significance. “*” means *p* < 0.05; “**” means *p* < 0.01).

Balancing aroma enrichment and fidelity, the optimal pretreatment combinations were identified as follows: a tea-to-water ratio of 1 g:10 mL, extraction at 30 °C for 30 min (hereafter referred to as 30 °C, 30 min), or a tea-to-water ratio of 1 g:10 mL extraction at 30 °C for 45 min (hereafter referred to as 30 °C, 45 min). Cosine similarity and correlation coefficients are commonly used as quantitative indicators of similarity, with values closer to 1 indicating higher similarity [7]. To validate the feasibility of these conditions, sensory fidelity was re-evaluated. As shown in Figure 5, the aroma profiles of infusions brewed for 4 min closely resembled those treated under high-fidelity extraction conditions. For the 30 °C, 30 min condition, cosine similarity scores were 0.991 (pure water system) and 0.983 (ionized water system), with correlation coefficients of 0.974 ** (pure water system) and 0.878 * (ionized water system). For the 30 °C, 45 min condition, cosine similarity scores were 0.983 (pure water system) and 0.979 (ionized water system), with correlation coefficients of 0.951 ** (pure water system) and 0.822 * (ionized water system). These results confirm the high sensory fidelity of the selected conditions.

### 3.3. Feasibility Analysis of Pretreatment Methods for Authentic Representation of Tea Infusion Aroma

#### 3.3.1. Technical Evaluation of Aroma Extraction Methods

To evaluate the stability of high-fidelity methods, an internal standard peak was used as the monitoring peak. The relative standard deviation (RSD) of retention time and peak area for the internal standard substance were calculated under high-fidelity methods with three replicates each. According to the established criteria, a method is considered reproducible if the RSD of retention time is ≤1.5% [7] and the RSD of peak area is ≤25% [27]. The results showed that under the 30 °C, 30 min condition, the RSD for retention time (RSD-RT) was 0.22% and the RSD for peak area (RSD-PA) was 6.95% in pure water, while the RSD-RT was 0.72% and the RSD-PA was 19.10% in ionized water. Under the 30 °C, 45 min condition, the RSD-RT was 0.23% and the RSD-PA was 2.06% in pure water, while the RSD-RT was 0.35% and the RSD-PA was 13.82% in ionized water. These findings indicated that the high-fidelity method exhibited excellent stability and reproducibility. Recoveries were determined by comparing the analytical results of spiked samples with their nominal concentrations. The method recovery rate of HS-SPME was demonstrated to range from 84% to 119% [30], indicating its capability for accurate determination of aroma compounds.

#### 3.3.2. Analysis of Method Application Effectiveness

To assess the practical application of the high-fidelity methods, tea infusions were prepared at a tea-to-water ratio of 1 g:10 mL under three extraction conditions: 30 °C for 30 min, 30 °C for 45 min, and 60 °C for 60 min, using both ionized water and pure water. Additionally, ionized water diluted to half its original TDS concentration (from 500 mg/L to 250 mg/L) was tested under the 30 °C, 45 min condition to evaluate the method’s discriminative power for different ion water.

**Figure 6:**
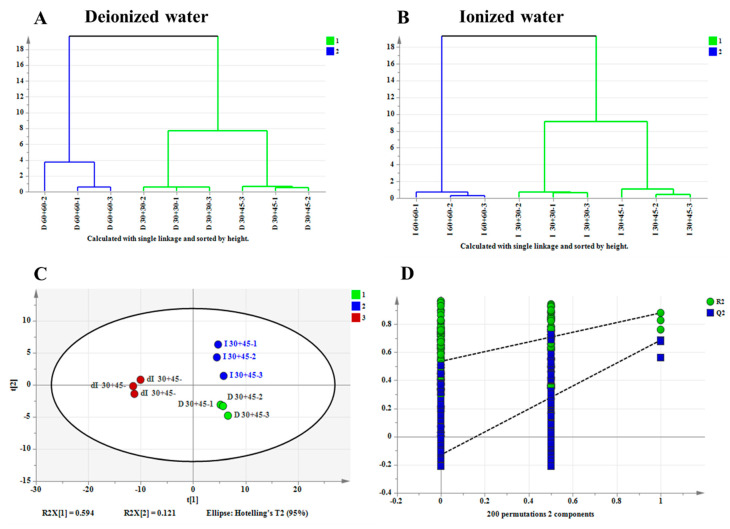
Comparative statistical analysis of aroma profiles in tea infusion under different extraction conditions (D = pure water; I = ionized water; dI = diluted ionized water. Numeric codes indicate: temperature + time-repetition e.g., D 60+60−1 = first replicate of pure water extraction at 60 °C for 60 min. The numbers 1, 2, 3, etc., represent categories for statistical classification.). (**A**) HCA of aroma components in pure water brewing system; (**B**) HCA of aroma components in ionized water brewing system; (**C**) PLS-DA of aroma components from different water types at 30 °C/45 min extraction; (**D**) Cross-validation plot by 200 permutation tests.

A total of 129 aroma compounds were identified across all pretreatment methods (Table S1). Using aroma component content as the dependent variable and different extraction conditions as independent variables, hierarchical clustering analysis (HCA) was performed. As shown in Figure 6A,B, for both pure water and ionized water systems, the aroma profiles under the 30 °C, 30 min and 30 °C, 45 min conditions clustered together, while those under the 60 °C, 60 min condition formed a separate cluster. This further confirmed the distinct aroma quality differences between high-temperature and long-duration conditions and high-fidelity conditions.

Using the content of aroma components as the dependent variable and three water types (deionized water, ionized water, and diluted ionized water) under the conditions of 30 °C and 45 min as independent variables, a Partial Least Squares Discrimination Analysis (PLS-DA) was performed. Figure 6C demonstrates that the aroma profiles of tea brewed with the three different ionic-strength waters at 30 °C for 45 min were clearly separated into three distinct clusters without overlap, indicating that the high-fidelity pretreatment method can effectively discriminate aroma characteristics influenced by water quality. The PLS-DA model exhibited satisfactory predictability, with cross-validation parameters R^2^_X_ = 0.715, R^2^_Y_ = 0.922, Q^2^ = 0.788, all exceeding the threshold of 0.5 [31]. Furthermore, the 200-iteration permutation test (shown in Figure 6D) confirmed the robustness of the model, as the intercept of the Q2 regression line with the vertical axis was below zero, demonstrating no overfitting and a reliable predictive performance [32].

## 4. Discussion

Tea aroma evaluation typically combines hot sniffing (60–80 °C), warm sniffing (25–55 °C), and cold sniffing (<25 °C) [26]. Hot sniffing captures high-boiling-point volatiles and off-odors but may impair olfactory sensitivity due to thermal stimulation. Cold sniffing assesses aroma persistence after cooling, while warm sniffing demonstrates superior evaluation accuracy, offers higher accuracy for evaluating dominant aroma characteristics and harmony. Thus, 30–60 °C represents the critical temperature range for aroma quality assessment and extraction parameter selection. In aroma recombination studies, solutions are often equilibrated at 25 °C [33,34] for olfactory validation, as no further aroma changes occur at this temperature. However, during HS-SPME extraction, prolonged high-temperature exposure may alter aroma profiles—a previously overlooked issue. Our methodology was developed by synthesizing the temperature for aroma evaluation while ensuring maximal preservation of aroma integrity during extraction and testing both pure and ionized water systems, ensuring methodological accuracy, specificity, and innovation. Validation tests confirmed that ionic systems exhibited lower stability than pure water, suggesting greater susceptibility to extraction conditions and underscoring the need for optimized protocols when analyzing ionized-water-infused aromas. However, in both ionic aqueous and pure water systems, extraction at 30 °C was found to minimize alterations in the aromatic profile before and after extraction, thereby representing the optimal temperature for screening high-fidelity extraction conditions.

Lin et al. investigated the aroma characteristics of Longjing tea using HS-SPME-GC-MS with extraction conditions of 60 °C for 60 min, identifying 10 key compounds significantly contributing to its aroma quality [14]. However, this study did not optimize the extraction parameters. Liu employed the HS-SPME-GC-MS methodology to compare different extraction temperatures (25, 35, 45, 55 °C) and durations (20, 40, 60, 80 min) for green tea infusion, concluding that 45 °C for 60 min provided optimal recovery of typical aroma components [35]. Notably, this work did not consider aroma fidelity as a factor, resulting in different optimal conditions from those identified in our current study. Our research group’s previous analysis of Longjing tea infusion aroma adopted high-fidelity extraction conditions (30 °C, 30 min) [23], which led, however, to a relatively low content of aromatic compounds. The present study reveals that while the tea-to-water ratio positively affects aroma enrichment, it has minimal impact on fidelity. Therefore, increasing the brewing ratio could be strategically employed to enhance extraction efficiency when sample quantity or budget permits, without compromising aroma authenticity. An extraction duration of 30–45 min was determined to be the safe window for preserving genuine aroma characteristics, and this can be adjusted for experimental efficiency. Notably, under high-temperature, long-duration conditions (60 °C, 60 min), ionized water systems exhibited higher total aroma enrichment than pure water but with intensified off-flavors. This phenomenon could be leveraged in mechanistic studies to amplify ionic effects and identify key odorants influenced by mineral content.

Although the high-fidelity conditions successfully preserved the authentic aroma profile and enabled precise quantification for aroma recombination experiments, the lower extraction yield compared to high-temperature/long-duration methods may obscure subtle differences in aroma content caused by varying ionic content. This study demonstrated the method’s capability to distinguish aroma profiles from water quality with a 250 mg/L TDS difference under 30 °C/45 min extraction conditions, but did not determine the minimum detectable TDS threshold that would affect aroma composition—a limitation of this research and a direction for future research.

## 5. Conclusions

An appropriate aroma extraction method is critical for accurate analysis of tea infusion aroma compounds. As for Longjing tea, high-temperature prolonged extraction conditions alter aroma profiles significantly. Extraction temperature emerged as the most critical factor in preserving the authentic tea aroma profile. Optimal conditions employing a 1 g:10 mL tea-to-water ratio at 30 °C for 30/45 min extraction duration were established to maximize aroma fidelity and enrichment. Compared to deionized water, ionized water exhibited markedly greater sensitivity to variations in extraction parameters. The developed protocol enabled discrimination of water quality-dependent aroma signatures at ΔTDS (Total Dissolved Solids) = 250 mg/L. This study provides a technical foundation for investigating the impact of water quality on genuine volatile compounds in green tea infusion.

## Figures and Tables

**Table 1:** Optimal design of aroma extraction conditions.

Influencing Factors	Extraction Condition Combinations
concentration of tea infusion	1 g:50 mL tea-to-water ratio, 30 °C for 30 min
	1 g:20 mL tea-to-water ratio, 30 °C for 30 min
	1 g:10 mL tea-to-water ratio, 30 °C for 30 min
extraction temperature	1 g:50 mL tea-to-water ratio, 30 °C for 30 min
	1 g:50 mL tea-to-water ratio, 45 °C for 30 min
	1 g:50 mL tea-to-water ratio, 60 °C for 30 min
extraction time	1 g:50 mL tea-to-water ratio, 30 °C for 30 min
	1 g:50 mL tea-to-water ratio, 30 °C for 45 min
	1 g:50 mL tea-to-water ratio, 30 °C for 60 min

## Data Availability

The original contributions presented in the study are included in the article/Supplementary Materials. Further inquiries can be directed to the corresponding authors.

## Supplementary Materials

The following supporting information can be downloaded at https://www.mdpi.com/article/10.3390/foods14162759/s1, Table S1: Information of 129 compounds identified in tea infusion.
