# Supplementary material for: Development of Pretreatment Approaches for Authentic Representation of Tea Infusion Aroma
Source: Foods. 2025 Aug 8;14(16):2759. doi: 10.3390/foods14162759 (PMC12385594; doi:10.3390/foods14162759)
Supplement: Supplementary file 1 [file foods-14-02759-s001.zip › foods-3775626-supplementary.pdf]

Supporting Information for

Development of Pretreatment Approaches for Authentic Representation of Tea Infusion Aroma

Mingming Zhang [1,2,3], Zhihui Feng [1], Fang Wang [1], Jianxin Chen [1], Yifan Li [1],

Yuqiong Chen [2,*] and Junfeng Yin [1,*]

1. Key Laboratory of Tea Biology and Resource Untilization, Ministry of Agriculture, Tea Research Institute, Chinese Academy of Agricultural Sciences, Hangzhou 310008, China; zhangmingming@tricaas.com (M.Z.)

2. National Key Laboratory for Germplasm Innovation and Utilization for Horticultural Crops, College of Horticulture & Forestry Sciences, Huazhong Agricultural University, Wuhan 430070, China

3. Graduate School of Chinese Academy of Agricultural Sciences, Beijing 100081, China

* Correspondence: chenyq@mail.hzau.edu.cn (Y.C.); yinjf@tricaas.com (J.Y.)

Table S1. List of the 129 compounds information identified in tea infusion.

No	Compounds	CAS	RI		Relative content（ug/L）							
			cal	ref	D 30+30	I 30+30	D 30+45	I 30+45	dI 30+45	D 60+60	I 60+60	dI 60+60
1	Dimethyl ether	115-10-6	654	/	1.432±0.052	1.395±0.153	1.219±0.075	0.988±0.308	0.465±0.363	0.263±0.082	0.247±0.042	0.628±0.391
2	Propanal, 2-methyl-	78-84-2	660	662	0.007±0.001	0.009±0.012	0.089±0.003	0.088±0.004	0.041±0.005	0.014±0.003	0.015±0.007	0.014±0.002
3	Furan, 3-methyl-	930-27-8	670	646	0.052±0.003	0.022±0.004	0.065±0.001	0.026±0.004	0.146±0.039	0.013±0.003	0.004±0.002	0.004±0.001
4	Butanal, 3-methyl-	590-86-3	682	669	0.411±0.027	0.25±0.023	0.674±0.018	0.461±0.188	0.416±0.114	0.034±0.003	0.049±0.014	0.018±0.002
5	Benzene	71-43-2	684	667	1.865±0.3	1.336±0.021	3.085±0.048	3.616±0.242	2.998±0.704	0.279±0.068	0.32±0.096	0.076±0.016
6	Butanal, 2-methyl-	96-17-3	684	681	0.134±0.01	0.072±0.014	0.217±0.009	0.117±0.018	0.626±0.103	0.013±0.002	0.036±0.009	0.01±0.004
7	Pentanal	110-62-3	699	690	0.884±0.053	0.724±0.097	0.17±0.001	0.149±0.028	0.265±0.021	0.059±0.012	0.15±0.064	0.208±0.086
8	Furan, 2-ethyl-	3208-16-0	699	702	0.819±0.134	0.188±0.006	0.472±0.027	0.536±0.047	0.639±0.034	0.08±0.009	0.196±0.089	0.145±0.048
9	2,4-Dimethylfuran	3710-43-8	706	708	0.344±0.044	0.087±0.007	0.045±0.003	0.108±0.137	0.061±0.008	0.023±0.001	0.022±0.003	0.007±0.004
10	2-Butenal, 2-methyl-	1115-11-3	724	744	0.018±0.009	0.011±0.002	0.016±0.006	0.015±0.001	0.012±0.004	0.03±0.006	0.03±0.006	0.021±0.003
11	Toluene	108-88-3	743	760	0.509±0.013	0.271±0.198	0.53±0.013	0.594±0.04	0.776±0.039	0.428±0.311	0.59±0.028	0.147±0.009
12	3-Hexanone, 2-methyl-	7379-12-6	762	784	0.275±0.039	0.027±0.014	0.004±0.001	0.004±0.001	0.151±0.009	0.116±0.087	0.013±0.003	0.006±0.001
13	Furan, 2-methoxy-	25414-22-6	771	/	0.804±0.143	0.629±0.029	1.378±0.084	1.484±0.156	0.913±0.03	1.781±0.264	1.112±0.052	1.094±0.181
14	Furan, 2-ethyl-5-methyl-	1703-52-2	773	789	0.029±0.004	0.017±0.002	0.036±0.006	0.041±0.004	0.044±0.003	0.034±0.007	0.025±0.005	0.034±0.008
15	Hexanal	66-25-1	775	799	2.06±0.293	0.294±0.036	0.03±0.003	0.032±0.004	1.087±0.064	1.636±0.3	0.808±0.113	0.412±0.457
16	1H-Pyrrole, 3-ethyl-	1551-16-2	784	/	0.361±0.164	0.157±0.01	0.459±0.038	0.459±0.031	0.324±0.023	0.31±0.234	0.232±0.027	0.258±0.113
17	2-Pentenal, 2-methyl-	623-36-9	805	826	0.958±0.854	0.778±0.902	0.028±0.002	0.026±0.005	0.015±0.001	0.036±0.004	0.041±0.009	0.018±0.002
18	3-Hexen-1-ol, (E)-	928-97-2	834	855	0.055±0.019	0.005±0.005	0.156±0.013	0.17±0.019	0.262±0.019	1.017±0.176	0.635±0.04	0.553±0.057
19	Ethylbenzene	100-41-4	837	860	0.213±0.004	0.351±0.028	0.141±0.004	0.164±0.015	0.063±0.003	0.081±0.016	0.05±0.005	0.051±0.007

No	Compounds	CAS	RI		Relative content（ug/L）							
			cal	ref	D 30+30	I 30+30	D 30+45	I 30+45	dI 30+45	D 60+60	I 60+60	dI 60+60
20	Benzene, 1,3-dimethyl-	108-38-3	847	867	0.106±0.026	0.16±0.011	0.414±0.012	0.481±0.065	0.148±0.001	0.139±0.008	0.149±0.017	0.137±0.008
21	(S)-(+)-3-Methyl-1-pentanol	42072-39-9	851	/	0.005±0.002	0.004±0.002	0.046±0.004	0.047±0.002	0.057±0.008	0.223±0.007	0.119±0.021	0.112±0.021
22	n-Butyl ether	142-96-1	865	873	0.182±0.026	0.149±0.004	0.207±0.018	0.171±0.019	0.141±0.015	0.071±0.01	0.1±0.01	0.06±0.002
23	2-Heptanone	110-43-0	873	892	0.02±0.002	0.022±0.001	0.05±0.004	0.054±0.002	0.064±0.004	0.156±0.018	0.136±0.023	0.09±0.005
24	2-n-Butyl furan	4466-24-4	873	897	0.124±0.017	0.116±0.004	0.023±0.003	0.023±0.002	0.043±0.003	0.057±0.001	0.044±0.004	0.048±0.003
25	2-Propenoic acid, butyl ester	141-32-2	882	892	0.22±0.028	0.195±0.009	0.262±0.012	0.217±0.023	0.164±0.01	0.26±0.033	0.215±0.018	0.16±0.002
26	Heptanal	111-71-7	888	903	0.384±0.046	0.349±0.003	0.504±0.024	0.415±0.036	0.725±0.053	1.665±0.129	0.932±0.055	0.984±0.018
27	Propanoic acid, butyl ester	590-01-2	896	900	0.001±0.001	0.002±0.001	0.222±0.01	0.183±0.018	0.068±0.003	0.085±0.023	0.064±0.004	0.053±0.008
28	2,3,4-Trimethylpyrrole	3855-78-5	908	/	0.031±0.001	0.015±0.002	0.085±0.009	0.08±0.006	0.048±0.009	0.116±0.03	0.101±0.013	0.087±0.005
29	Hexanoic acid, methyl ester	106-70-7	914	929	0.001±0.001	0.001±0.001	0.091±0.008	0.091±0.003	0.015±0.002	0.024±0.004	0.025±0.004	0.016±0.001
30	Hexanal, 3,3-dimethyl-	55320-57-5	939	/	0.002±0.001	0.001±0.002	0.01±0.001	0.009±0.001	0.012±0.002	0.013±0.002	0.014±0.002	0.009±0.001
31	Benzene, propyl-	103-65-1	945	950	0.005±0.001	0.005±0.002	0.011±0.002	0.015±0.001	0.008±0.002	0.033±0.005	0.039±0.012	0.015±0.006
32	2-Heptanone, 6-methyl-	928-68-7	948	954	0.005±0.001	0.004±0.002	0.01±0.001	0.01±0.001	0.011±0.002	0.023±0.002	0.023±0.003	0.018±0.002
33	Benzaldehyde	100-52-7	955	960	0.026±0.023	0.031±0.006	0.169±0.016	0.173±0.001	0.13±0.013	0.773±0.094	0.503±0.014	0.694±0.071
34	Benzene, 1,2,4-trimethyl-	95-63-6	955	968	0.048±0.001	0.031±0.006	0.013±0.002	0.017±0.005	0.01±0.002	0.898±0.161	0.489±0.036	0.621±0.061
35	1-Heptanol	111-70-6	969	970	0.004±0.002	0.003±0.001	0.005±0.002	0.004±0.001	0.008±0.002	0.054±0.008	0.051±0.009	0.056±0.005
36	1-Octen-3-ol	3391-86-4	980	978	0.036±0.033	0.002±0.001	0.047±0.007	0.041±0.005	0.059±0.01	0.587±0.103	0.42±0.052	0.381±0.055
37	Tetraethyl silicate	78-10-4	984	/	0.001±0.001	0.001±0.001	0.084±0.007	0.086±0.006	0.025±0.005	1.679±0.146	0.045±0.008	0.039±0.002

No	Compounds	CAS	RI		Relative content（ug/L）							
			cal	ref	D 30+30	I 30+30	D 30+45	I 30+45	dI 30+45	D 60+60	I 60+60	dI 60+60
38	Butanoic acid, butyl ester	109-21-7	998	994	0.001±0.001	0.001±0.001	0.004±0.001	0.003±0.001	0.046±0.005	0.084±0.012	0.072±0.007	0.083±0.008
39	Hexanoic acid, ethyl ester	123-66-0	1002	1001	0.001±0.001	0.001±0.001	0.03±0.004	0.029±0.003	0.042±0.01	0.085±0.013	0.109±0.066	0.092±0.01
40	Octanal	124-13-0	1006	1004	0.001±0.001	0.001±0.001	0.07±0.006	0.054±0.011	0.14±0.009	0.55±0.009	0.32±0.019	0.357±0.041
41	2,4-Heptadienal, (E,E)-	4313-03-5	1014	1022	0.002±0.001	0.001±0.001	0.072±0.003	0.086±0.01	0.025±0.003	0.294±0.042	0.176±0.018	0.275±0.015
42	Acetic acid, hexyl ester	142-92-7	1017	1018	0.001±0.001	0.002±0.001	0.008±0.001	0.008±0.001	0.011±0.002	0.047±0.011	0.026±0.004	0.023±0.003
43	1,3-Cyclohexadiene, 1-methyl-4-(1-methylethyl)-	99-86-5	1019	1018	0.006±0.001	0.003±0.001	0.009±0.001	0.008±0.001	0.008±0.002	0.043±0.007	0.04±0.003	0.021±0.001
44	2-Hexen-1-ol, acetate, (E)-	2497-18-9	1009	1010	0.008±0.002	0.003±0.001	0.806±0.026	0.962±0.105	2.053±0.22	6.599±0.697	4.642±0.375	5.486±0.286
45	D-Limonene	5989-27-5	1033	1025	0.006±0.001	0.005±0.001	0.074±0.007	0.078±0.009	0.088±0.019	0.565±0.022	0.216±0.023	0.379±0.098
46	1-Hexanol, 2-ethyl-	104-76-7	1035	1031	0.005±0.002	0.005±0.004	0.011±0.003	0.03±0.007	0.012±0.003	0.147±0.016	0.13±0.027	0.067±0.003
47	Benzeneethanol, .beta.-ethenyl-	6052-63-7	1036	/	0.001±0.001	0.001±0.001	0.005±0.002	0.006±0.002	0.009±0.002	0.017±0.002	0.015±0.004	0.021±0.002
48	Benzene, iodo-	591-50-4	1037	1033	0.001±0.001	0.001±0.001	0.013±0.001	0.004±0.002	0.002±0.001	0.003±0.002	0.006±0.002	0.004±0.001
49	Cyclohexanone, 2,2,6-trimethyl-	2408-37-9	1039	1035	0.027±0.005	0.008±0.001	0.05±0.004	0.05±0.002	0.037±0.004	0.083±0.017	0.094±0.013	0.091±0.008
50	2,4,6-Trimethyl-1,3,6-heptatriene	24648-33-7	1042	/	0.006±0.002	0.005±0.001	0.013±0.002	0.013±0.001	0.015±0.003	0.059±0.009	0.119±0.011	0.05±0.005
51	3-Octen-2-one	1669-44-9	1044	1036	0.004±0.001	0.003±0.002	0.005±0.001	0.005±0.001	0.004±0.002	0.033±0.013	0.037±0.01	0.029±0.006
52	Benzeneacetaldehyde	122-78-1	1049	1042	0.007±0.001	0.004±0.002	0.253±0.031	0.191±0.01	0.052±0.015	1.138±0.158	1.389±0.246	1.089±0.12

No	Compounds	CAS	RI		Relative content（ug/L）							
			cal	ref	D 30+30	I 30+30	D 30+45	I 30+45	dI 30+45	D 60+60	I 60+60	dI 60+60
53	3-Formyl-4,5-dimethyl-pyrrole	1000145-89-7	1053	/	0.002±0.002	0.001±0.001	0.158±0.017	0.148±0.01	0.135±0.018	1.182±0.266	0.669±0.018	0.971±0.009
54	β-Ocimene	13877-91-3	1054	1031	0.007±0.002	0.005±0.001	0.047±0.003	0.046±0.003	0.124±0.011	0.268±0.024	0.264±0.019	0.236±0.01
55	4-Undecene, 3-methyl-, (Z)-	74645-87-7	1066	/	0.058±0.054	0.092±0.02	0.004±0.002	0.003±0.001	0.008±0.001	0.083±0.013	0.044±0.002	0.055±0.002
56	Decane, 4-methyl-	2847-72-5	1069	1060	0.064±0.014	0.042±0.007	0.003±0.001	0.002±0.001	0.005±0.001	0.012±0.001	0.013±0.004	0.011±0.003
57	Formic acid, octyl ester	112-32-3	1082	1109	0.115±0.022	0.126±0.008	0.021±0.007	0.016±0.003	0.048±0.006	0.866±0.077	0.361±0.283	0.627±0.041
58	Benzene, 2-ethyl-1,4-dimethyl-	1758-88-9	1083	1072	0.006±0.001	0.004±0.001	0.006±0.001	0.007±0.001	0.008±0.002	0.022±0.006	0.02±0.008	0.018±0.001
59	Pyrazine, 2,6-diethyl-	13067-27-1	1086	1078	0.012±0.011	0.001±0.001	0.025±0.002	0.024±0.002	0.011±0.002	0.216±0.042	0.245±0.048	0.236±0.017
60	Linalool	78-70-6	1114	1104	0.001±0.001	0.001±0.001	0.32±0.029	0.337±0.033	0.439±0.019	5.621±0.686	4.634±0.51	4.499±0.122
61	Nonanal	124-19-6	1119	1109	0.003±0.001	0.001±0.001	0.264±0.042	0.206±0.043	0.425±0.014	1.986±0.075	1.321±0.064	1.573±0.027
62	(E)-4,8-Dimethylnona-1,3,7-triene	19945-61-0	1126	1116	0.003±0.001	0.001±0.001	0.037±0.002	0.037±0.002	0.129±0.014	0.14±0.012	0.167±0.014	0.178±0.01
63	Benzene, 1,2,3,5-tetramethyl-	527-53-7	1130	1123	0.001±0.001	0.001±0.001	0.014±0.001	0.017±0.002	0.017±0.001	0.033±0.005	0.035±0.002	0.034±0.002
64	Benzene, 1,2-dichloro-4-methyl-	95-75-0	1131	1125	0.001±0.001	0.001±0.001	0.004±0.001	0.006±0.002	0.005±0.001	0.011±0.001	0.011±0.002	0.011±0.001
65	2,4,6-Octatriene, 2,6-dimethyl-	673-84-7	1141	1119	0.006±0.001	0.003±0.003	0.005±0.001	0.005±0.001	0.006±0.001	0.033±0.004	0.04±0.004	0.017±0.001
66	1,3,8-p-Menthatriene	18368-95-1	1143	1118	0.001±0.001	0.001±0.001	0.004±0.001	0.004±0.001	0.008±0.001	0.038±0.003	0.038±0.003	0.032±0.005
67	(R,S)-5-Ethyl-6-methyl-3E-hepten-2-one	57283-79-1	1155	1143	0.001±0.001	0.001±0.001	0.01±0.002	0.009±0.001	0.009±0.001	0.07±0.008	0.059±0.004	0.067±0.002

No	Compounds	CAS	RI		Relative content（ug/L）							
			cal	ref	D 30+30	I 30+30	D 30+45	I 30+45	dI 30+45	D 60+60	I 60+60	dI 60+60
68	cis-3-Hexenyl iso-butyrate	41519-23-7	1157	1142	0.06±0.004	0.044±0.012	0.012±0.002	0.013±0.002	0.035±0.004	0.136±0.01	0.112±0.011	0.123±0.002
69	Pyrazine, 3,5-diethyl-2-methyl-	18138-05-1	1168	1148	0.001±0.001	0.001±0.001	0.006±0.001	0.005±0.001	0.002±0.001	0.044±0.003	0.07±0.012	0.071±0.003
70	2-Nonenal, (E)-	18829-56-6	1175	1165	0.001±0.001	0.001±0.001	0.002±0.001	0.002±0.001	0.004±0.002	0.101±0.008	0.047±0.005	0.068±0.003
71	1-Nonanol	143-08-8	1187	1174	0.001±0.001	0.001±0.001	0.008±0.003	0.006±0.004	0.003±0.001	0.193±0.03	0.149±0.043	0.169±0.008
72	1,4-Benzenedicarboxaldehyde	623-27-8	1188	1166	0.001±0.001	0.001±0.001	0.002±0.001	0.002±0.001	0.003±0.001	0.044±0.004	0.036±0.007	0.054±0.003
73	(3R,6S)-2,2,6-Trimethyl-6-vinyltetrahydro-2H-pyran-3-ol	39028-58-5	1190	1173	0.004±0.001	0.001±0.001	0.005±0.001	0.006±0.002	0.009±0.001	0.739±0.061	0.433±0.042	0.601±0.072
74	Terpinen-4-ol	562-74-3	1195	1185	0.001±0.001	0.001±0.001	0.002±0.001	0.002±0.001	0.001±0.001	0.034±0.006	0.019±0.001	0.023±0.002
75	Naphthalene	91-20-3	1196	1183	0.001±0.001	0.001±0.001	0.027±0.004	0.027±0.002	0.055±0.01	0.382±0.026	0.453±0.04	0.549±0.043
76	Isophthalaldehyde	626-19-7	1197	/	0.001±0.001	0.001±0.001	0.004±0.001	0.003±0.001	0.003±0.002	0.026±0.003	0.022±0.001	0.034±0.006
77	Butanoic acid, 3-hexenyl ester, (E)-	53398-84-8	1202	1185	0.06±0.016	0.021±0.008	0.75±0.108	0.846±0.164	2.367±0.141	9.832±0.332	9.107±0.911	9.623±0.211
78	Methyl salicylate	119-36-8	1205	1206	0.001±0.001	0.001±0.001	0.068±0.018	0.07±0.017	0.128±0.015	2.525±0.496	1.591±0.362	2.412±0.133
79	α-Terpineol	98-55-5	1210	1193	0.001±0.001	0.001±0.001	0.003±0.001	0.003±0.001	0.003±0.001	0.081±0.014	0.057±0.007	0.076±0.003
80	Octanoic acid, ethyl ester	106-32-1	1212	1197	0.006±0.006	0.002±0.003	0.008±0.003	0.011±0.007	0.008±0.001	0.064±0.009	0.178±0.021	0.04±0.006

No	Compounds	CAS	RI		Relative content（ug/L）							
			cal	ref	D 30+30	I 30+30	D 30+45	I 30+45	dI 30+45	D 60+60	I 60+60	dI 60+60
81	1,3-Cyclohexadiene-1-carboxaldehyde, 2,6,6-trimethyl-	116-26-7	1213	1198	0.006±0.001	0.004±0.001	0.041±0.005	0.041±0.003	0.027±0.004	0.282±0.045	0.213±0.014	0.245±0.005
82	Dodecane	112-40-3	1216	1200	0.031±0.011	0.037±0.006	0.009±0.004	0.065±0.057	0.043±0.062	1.66±0.544	1.061±0.101	0.069±0.018
83	Decanal	112-31-2	1222	1208	0.058±0.017	0.01±0.004	0.084±0.007	0.329±0.447	2.237±0.327	0.835±0.133	0.871±0.055	8.747±0.703
84	1-Cyclohexene-1-carboxaldehyde, 2,6,6-trimethyl-	432-25-7	1233	1224	0.001±0.001	0.001±0.001	0.029±0.004	0.027±0.001	0.025±0.004	0.238±0.026	0.192±0.008	0.23±0.002
85	1-Phenyl-2-butanone	1007-32-5	1237	/	0.001±0.001	0.001±0.001	0.012±0.001	0.005±0.005	0.002±0.001	0.336±0.036	0.139±0.019	0.302±0.076
86	cis-3-Hexenyl-.alpha.-methylbutyrate	53398-85-9	1245	1233	0.001±0.001	0.001±0.001	0.281±0.122	0.294±0.111	1.076±0.062	3.95±0.059	3.024±0.1	3.932±0.054
87	6-Undecanone	927-49-1	1253	1249	0.001±0.001	0.001±0.001	0.007±0.002	0.006±0.002	0.01±0.002	0.51±0.077	0.377±0.02	0.514±0.029
88	Benzene, 1,3-bis(1,1-dimethylethyl)-	1014-60-4	1261	1249	0.001±0.001	0.001±0.001	0.001±0.001	0.002±0.001	0.001±0.001	0.003±0.001	0.024±0.004	0.003±0.001
89	2,6-Octadien-1-ol, 3,7-dimethyl-, (Z)-	106-25-2	1265	1255	0.001±0.001	0.001±0.001	0.003±0.001	0.012±0.013	0.06±0.005	6.15±0.141	4.369±0.527	4.525±0.339
90	1-Cyclohexene-1-acetaldehyde, 2,6,6-trimethyl-	472-66-2	1268	1253	0.001±0.001	0.001±0.001	0.008±0.002	0.008±0.001	0.007±0.001	0.046±0.002	0.046±0.005	0.052±0.005
91	2-Decenal, (E)-	3913-81-3	1275	1268	0.001±0.001	0.001±0.001	0.001±0.001	0.001±0.001	0.002±0.001	0.048±0.003	0.025±0.002	0.035±0.003
92	Citral	5392-40-5	1265	1272	0.001±0.001	0.001±0.001	0.005±0.004	0.007±0.012	0.06±0.005	2.941±0.765	3.857±0.56	4.53±0.333
93	1-Decanol	112-30-1	1285	1269	0.001±0.001	0.001±0.001	0.001±0.001	0.001±0.001	0.001±0.001	0.056±0.003	0.041±0.005	0.045±0.005

No	Compounds	CAS	RI		Relative content（ug/L）							
			cal	ref	D 30+30	I 30+30	D 30+45	I 30+45	dI 30+45	D 60+60	I 60+60	dI 60+60
94	n-Valeric acid cis-3-hexenyl ester	35852-46-1	1295	1270	0.01±0.004	0.001±0.001	0.002±0.001	0.002±0.001	0.004±0.001	0.04±0.002	0.044±0.002	0.038±0.002
95	Tridecane	629-50-5	1309	1300	0.003±0.001	0.002±0.001	0.003±0.003	0.001±0.001	0.002±0.001	0.071±0.097	0.013±0.003	0.017±0.004
96	Undecanal	112-44-7	1315	1305	0.001±0.001	0.001±0.001	0.003±0.002	0.004±0.002	0.004±0.002	0.048±0.002	0.075±0.01	0.045±0.017
97	(Z)-Hex-3-enyl (E)-2-methylbut-2-enoate	1000373-73-0	1325	/	0.069±0.001	0.02±0.003	0.006±0.004	0.006±0.003	0.007±0.002	0.16±0.015	0.184±0.008	0.167±0.009
98	1, 1, 5-Trimethyl-1, 2-dihydronaphthalene	1000357-25-8	1345	/	0.001±0.001	0.001±0.001	0.028±0.007	0.026±0.005	0.011±0.004	0.072±0.006	0.062±0.003	0.067±0.006
99	Hexanoic acid, 3-hexenyl ester, (Z)-	31501-11-8	1362	1369	0.071±0.001	0.026±0.002	0.05±0.043	0.151±0.019	1.597±0.073	7.643±0.256	6.009±0.317	7.772±1.084
100	cis-3-Hexenyl cis-3-hexenoate	61444-38-0	1363	1388	0.071±0.001	0.025±0.002	0.05±0.043	0.151±0.019	0.088±0.016	0.994±0.087	0.673±0.008	0.991±0.114
101	2-Cyclopenten-1-one, 3-methyl-2-(2-pentenyl)-, (Z)-	488-10-8	1369	1389	0.001±0.001	0.001±0.001	0.005±0.002	0.005±0.001	0.022±0.006	1.415±0.182	1.736±0.284	2.045±0.29
102	Tetradecane	629-59-4	1373	1400	0.001±0.001	0.001±0.001	0.003±0.003	0.002±0.001	0.002±0.001	0.019±0.003	0.015±0.004	0.025±0.002
103	1H-3a,7-Methanoazulene, 2,3,4,7,8,8a-hexahydro-3,6,8,8-tetramethyl-, [3R-(3.alpha.,3a.beta.,7.beta.,8a.alpha.)]-	469-61-4	1388	1416	0.001±0.001	0.001±0.001	0.001±0.001	0.002±0.001	0.001±0.001	0.03±0.009	0.049±0.014	0.03±0.008

No	Compounds	CAS	RI		Relative content（ug/L）							
			cal	ref	D 30+30	I 30+30	D 30+45	I 30+45	dI 30+45	D 60+60	I 60+60	dI 60+60
104	α-Ionone	127-41-3	1394	1424	0.001±0.001	0.001±0.001	0.002±0.001	0.002±0.001	0.001±0.001	0.057±0.009	0.055±0.005	0.059±0.004
105	1H-3a,7-Methanoazulene, octahydro-3,8,8-trimethyl-6-methylene-, [3R-(3.alpha.,3a.beta.,7.beta.,8a.alpha.)]-	546-28-1	1401	1428	0.001±0.001	0.001±0.001	0.001±0.001	0.001±0.001	0.001±0.001	0.137±0.168	0.043±0.011	0.032±0.014
106	5,9-Undecadien-2-one, 6,10-dimethyl-, (Z)-	3879-26-3	1431	1427	0.001±0.001	0.001±0.001	0.001±0.001	0.001±0.001	0.001±0.001	0.213±0.021	0.097±0.01	0.152±0.034
107	2,5-Cyclohexadiene-1,4-dione, 2,6-bis(1,1-dimethylethyl)-	719-22-2	1451	1472	0.001±0.001	0.001±0.001	0.001±0.001	0.005±0.003	0.001±0.001	0.002±0.002	0.046±0.019	0.001±0.001
108	1-Dodecanol	112-53-8	1469	1471	0.001±0.001	0.001±0.001	0.001±0.001	0.001±0.001	0.001±0.001	0.042±0.014	0.055±0.04	0.03±0.004
109	3-Buten-2-one, 4-(2,6,6-trimethyl-1-cyclohexen-1-yl)-	14901-07-6	1476	1491	0.001±0.001	0.001±0.001	0.007±0.003	0.007±0.003	0.007±0.001	0.604±0.07	0.525±0.014	0.633±0.024
110	2,4-Di-tert-butylphenol	96-76-4	1511	1502	0.001±0.001	0.001±0.001	0.002±0.001	0.004±0.004	0.004±0.001	0.234±0.026	0.165±0.023	0.142±0.019
111	Tridecanal	10486-19-8	1517	1517	0.001±0.001	0.001±0.001	0.001±0.001	0.001±0.001	0.001±0.001	0.028±0.028	0.028±0.015	0.178±0.008
112	Naphthalene, 1,2,4a,5,8,8a-hexahydro-4,7-dimethyl-1-(1-	523-47-7	1529	1519	0.001±0.001	0.001±0.001	0.04±0.013	0.026±0.002	0.079±0.005	4.677±0.81	1.708±0.253	2.746±0.132

No	Compounds	CAS	RI		Relative content（ug/L）							
			cal	ref	D 30+30	I 30+30	D 30+45	I 30+45	dI 30+45	D 60+60	I 60+60	dI 60+60
	methylethyl)-, [1S-(1.alpha.,4a.beta.,8a.alpha.)]-											
113	Naphthalene, 1,2,3,4-tetrahydro-1,6-dimethyl-4-(1-methylethyl)-, (1S-cis)-	483-77-2	1534	1519	0.001±0.001	0.001±0.001	0.06±0.014	0.057±0.005	0.128±0.047	5.659±0.548	2.267±0.067	3.582±0.624
114	Naphthalene, 1,2,3,4,4a,7-hexahydro-1,6-dimethyl-4-(1-methylethyl)-	16728-99-7	1547	1533	0.001±0.001	0.001±0.001	0.003±0.003	0.003±0.002	0.008±0.003	0.558±0.067	0.22±0.02	0.319±0.041
115	1,6,10-Dodecatrien-3-ol, 3,7,11-trimethyl-	7212-44-4	1578	1558	0.001±0.001	0.001±0.001	0.001±0.001	0.001±0.001	0.002±0.002	0.183±0.02	0.268±0.084	0.142±0.038
116	4-Isopropyl-6-methyl-1-methylene-1,2,3,4-tetrahydronaphthalene	50277-34-4	1583	1566	0.001±0.001	0.001±0.001	0.001±0.001	0.001±0.001	0.003±0.001	0.146±0.023	0.054±0.009	0.148±0.028
117	2,2,4-Trimethyl-1,3-pentanediol diisobutyrate	6846-50-0	1608	1587	0.001±0.001	0.001±0.001	0.002±0.002	0.009±0.008	0.001±0.001	0.024±0.007	0.25±0.22	0.036±0.014
118	Phenol, 2,4,6-tri-tert-butyl-	732-26-3	1611	/	0.001±0.001	0.001±0.001	0.001±0.001	0.001±0.001	0.001±0.001	0.001±0.001	0.008±0.001	0.001±0.001
119	Tetradecanal	124-25-4	1641	1617	0.001±0.001	0.001±0.001	0.001±0.001	0.001±0.001	0.005±0.004	0.012±0.002	0.01±0.002	0.011±0.001
120	Cedrol	77-53-2	1641	1611	0.001±0.001	0.001±0.001	0.001±0.001	0.001±0.001	0.001±0.001	0.088±0.004	0.06±0.004	0.055±0.014

No	Compounds	CAS	RI		Relative content（ug/L）							
			cal	ref	D 30+30	I 30+30	D 30+45	I 30+45	dI 30+45	D 60+60	I 60+60	dI 60+60
121	α-Corocalene	20129-39-9	1644	1623	0.001±0.001	0.001±0.001	0.001±0.001	0.001±0.001	0.002±0.001	0.211±0.038	0.077±0.014	0.125±0.016
122	2,6-Bis(1,1-dimethylethyl)-4-(1-oxopropyl)phenol	14035-34-8	1647	1635	0.001±0.001	0.001±0.001	0.002±0.001	0.004±0.003	0.003±0.002	0.011±0.003	0.018±0.01	0.008±0.003
123	Ylangenal	41610-68-8	1666	1674	0.001±0.001	0.001±0.001	0.001±0.001	0.001±0.001	0.001±0	0.057±0.005	0.062±0.011	0.064±0.006
124	Tetradecanoic acid, ethyl ester	124-06-1	1813	1794			0.001±0.001	0.001±0.001	0.001±0.001	0.003±0.002	0.004±0.004	0.002±0.002
125	Cyclopenta[g]-2-benzopyran, 1,3,4,6,7,8-hexahydro-4,6,6,7,8,8-hexamethyl-	1222-05-5	1865	1850			0.001±0.001	0.001±0.001	0.001±0	0.017±0.005	0.014±0.002	0.016±0.003
126	Phthalic acid, hept-4-yl isobutyl ester	1000356-78-3	1875	/			0.003±0.004	0.009±0.007	0.002±0.001	0.708±0.046	0.664±0.009	0.5±0.012
127	Nonadecane	629-92-5	1908	1900			0.001±0.001	0.001±0.001	0.001±0.001	0.001±0.001	0.003±0.001	0.019±0.002
128	Dibutyl phthalate	84-74-2	1956	1970			0.001±0.001	0.003±0.002	0.003±0.001	0.353±0.406	0.406±0.06	0.201±0.146
129	Hexadecanoic acid, ethyl ester	628-97-7	1984	1994			0.001±0.001	0.001±0.001	0.001±0.001	0.009±0.001	0.002±0.001	0.002±0.001

Note: Retention index referred to the literature value was founded in the website: https://webbook.nist.gov/chemistry/. ‘/’, the RI was not founded. blank, the compound was not detected in the sample. D = pure water; I = ionized water; dI = diluted ionized water. Numeric codes indicate: temperature + time. e.g., D 60+60= pure water, extraction at 60°C for 60 min. All extraction conditions employed a consistent tea-to-water ratio of 1g:10 mL.
